# Supplementary material for: Anne O’Tate: Value-added PubMed search engine for analysis and text mining
Source: PLoS One. 2021 Mar 8;16(3):e0248335. doi: 10.1371/journal.pone.0248335 (PMC7939269; doi:10.1371/journal.pone.0248335)
Supplement: Attachment — Submitted filename: Response to Reviewers 091320.docx [file pone.0248335.s001.docx]

**Response to Reviewers**

**Reviewer #1:** The authors present the recent functional updates of Anne O’Tate, a web tool that enhances PubMed search engine to provide users with several additional functions to analyze the search results. This manuscript if published will help publicize the tool and serve as a tutorial for users. I only have a couple minor comments:

1. Adding an introduction to MEDLINE and MeSH terms will help readers who are not familiar with these terms to understand better on the methodologies and output of the tool.

We have now added more information about what MeSH terms are and what MEDLINE is, including a reference.

2. Is the ranking of retrieved results taken into consideration (weighted differently) in summarizing on Topics, Authors, Affiliations etc.?

We have stated that in the initial query results, articles are displayed in reverse chronological order, not by predicted relevance. We also state for each button how articles are ranked – sometimes by frequency, sometimes by importance scores which have been defined in our previous publication.

**Reviewer #2:** The authors present a nice overview of their Anne O’Tate tool for querying the PubMed/Medline database. All functions were described with details, as well as the methods behind them. However, it is still not clear which functions are actually new and I miss good examples (use cases) for many of the functions proposed in the tool.

We have now added use cases to the paper and discussed them in some detail. Also, we have now clarified in Results that we have added one asterisk to indicate buttons that have been modified substantially since our previous publication, and two asterisks to indicate entirely new buttons.

Major review:

- The authors implemented many functions in the tool, but I miss a real use case which could demonstrate that some (interesting) insights could only have been learned by using some of the proposed function and not with Pubmed itself or other available tool.

Done.

- Indeed, I miss short discussion of other available tool, since many tools are indeed available, and how these compared to Anne O’Tate.

In the Discussion we cite several reviews that have discussed similar tools in detail, including a comparison to Anne O’tate.

Minor review:

- The abstract does not mention what is new in the tool in comparison to its older versions.

We have now extensively rewritten the abstract to include the original set of buttons and to emphasize specific new buttons that have been added.

- The introduction does not cite how many visits (e.g., per day, or in total) the tool has had in the last years, nor whether it has been helpful to support the research from others (e.g., publications that cited Anne O'Tate). I also miss a short overview of what are the new features that will be describe in this new publication.

We have now added text in the Introduction pointing out several groups who have reviewed Anne’s performance (before the recent improvements) and compared it to other existing tools. The added text to the abstract should provide the requested overview of new features up front.

- I am not sure whether some of functions are indeed helpful. For instance, by using raw frequency, the most popular surnames will always be on the top of the author list. It was also not clear to me whether the author count could be indeed helpful. I miss examples or use cases on how these functions might help the search.

We have now added a use case for the Author Count function, and added more description of the Author Names and how to use them. We are now displaying Authors by lastname firstinitial rather than simply by lastname, which will help with disambiguation somewhat. We also alert the reader that we have disambiguated authors on papers in a separate project which is being released publicly through 2019 (even if the most recent papers are not yet linked directly to Anne yet).

- Further, for the affiliation function, it is indeed confusing, since it is a mix of countries, cities, departments and universities. Further, “USA” appeared twice: “USA” and “USA.”

We have now added further text to describe how to use the Affiliation chunks.

- I found that the example of an earthquake in Puerto Rico was not suitable (off topic) since the manuscript so far had been showing the use case of treatment for Alzheimer disease.

We have now changed the example to be consistent.

- “Mine the Gap!” is indeed a creative function, but top results were pairs that probably do not make sense together, e.g. “Caregivers::Mice, Transgenic”, even though some might be interesting, e.g., “Mice, Transgenic::Surveys and Questionnaires”. Again, a good use case showing its utility would help.

We have now added more description and a use case here (although this tool is fully described in detail in a separate publication that is now a few years old).

**Reviewer #3:** This study presents the recent updates of Anne O’Tate, a web server provides additional analytics to PubMed query results. It introduces primary features such as important words, important phrases, topic clustering and many others. Altogether it provides rich information over the retrieved pmids. Please find my comments below.

I evaluated the system using the query “covid-19 diabetes” and found a few issues:
(1) The relevance of top k pmids is of concern. The top 5 retrieved PMIDs were 32634827, 32634717, 32634716, 32634459, 32633728. They do have the term ‘diabetes’, but diabetes is not the main topic, so I am not sure why they are ranked at the top. Comparatively, the top 5 pmids from both PubMed and LitCovid are much more relevant – the content is directly on diabetes. The paper claims that it passes the query to PubMed, but the retrieved results are significantly different. And this is not because of publication dates (the study uses the PubMed data up to May) as some of the top retrieved pmids from PubMed were published before May. The retrieved pmids are critical since all the features are based on them. I suspect there are some issues for query expansion and translation, before passing to PubMed.

As mentioned above and stated in the paper, the retrieved articles are listed in reverse chronological order, NOT in predicted relevance. But beyond that, it is important to note that PubMed changed their public interface in a very major way this spring, rather suddenly, so that their new interface works very differently than it had for many years. We built our tool to be compatible with the old PubMed interface, and our tool is not altered at all – however, it does not always coincide with the way that PubMed displays results, depending on user settings. We have now added text to the Discussion to alert users to the possible discrepancy – which does not affect the utility of our tool in any way.

(2) The results of ‘Affiliations’ for this query is confusing. The top affiliations are a mixed of countries (e.g., China), cities (e.g., Milan), universities (e.g., Huazhong University of Science and Technology), and departments (e.g., Department of Medicine). So what exactly is an affiliation? Huazhong University of Science and Technology is a university in China. Why showing them separately? It would be much better to represent in a hierarchical manner. Post-processing the Affiliation field of PubMed records is essential. The current results are confusing and not informative.

As mentioned above, we have now clarified that the method of displaying Affiliations by chunks may be initially confusing and give hints how to use the results. We also now cite the work of Torvik who has developed a geographical mapping tool for PubMed articles, which may be used as an alternative to our Affiliations button.

(3) The results of ‘Clustered by topic’ also have problems. When clicking the “Most recent articles” cluster, it shows an error message “Request-URI Too Long”. Also, the topic “AMP-Activated Protein Kinases” has only 1 pmid, why this is considered as a topic?

The reviewer chose a very unusual query – covid-19 is an extremely new topic so almost all of the papers lack MeSH terms which are employed for clustering. The set of papers actually clustered is thus quite small, and we always divide the set into 12 partitions, which explains why AMP-activated protein kinases was included. Normally, the bulk of retrieved articles are not extremely new, so the bulk of articles are clustered. As for the URI-error, this is a limitation of entering PMIDs into the PubMed query box, which we discussed in the Methods section, and we have now addressed the issue by fetching the records another way.

(4) For the results of ‘important words’, somehow ‘PubMed’ is listed as an important word.

This is not a bug. PubMed IS mentioned in many covid-19 articles, e.g., systematic reviews where they say that they searched PubMed for relevant articles.

In addition, while it introduces many features, there is no evaluation detailed in the paper. Some of the adopted methods were published a while ago; also the study cited a few methods that are in preparation without giving details. The effectiveness of these methods is unclear. The study should provide a substantial evaluation of the methods and compares with existing methods as baselines.

We have now given several additional use cases which should be persuasive that the tool has value for biomedical users. The algorithmic aspects are described and evaluated in detail in previous publications, and the newest ones (e.g. predicted publication types) will be described in detail in separate publications. We are confident that the results that we give are “correct” and transparent. However, the value of Anne O’Tate is not so much the uniqueness of the algorithms but the fact that they are packaged together into one seamless integrated platform. There is no comparable tool.

Minor comments:
(1) Please provide an API which supports batch processing.

The reviewer is asking for us to build an extensive option which is not part of the system, and which no one has ever asked us to build before. I don’t think that we can do this as a condition of revising the paper, although if there is serious user interest we would consider it.

(2) What is the usage of the system, and how often does the system update?

We have not kept track of recent usage since we moved the web server to a new server machine and expanded memory. In any case, we do not expect that user load will be a significant factor in performance, even if the user base expands after this paper is published. The Anne “system” is a set of web-based tools and does not have a formal release (unlike software). The updates to the system are described in this paper, so you could think of this paper as describing Anne 2.0.

(3) For line 207-209, the font is different.

Fixed.

**Reviewer #4:** This paper is a brief introduction to Anne O'Tate, which adds additional functionalities to PubMed and intends to help PubMed users.
Although some interesting gadgets are proposed and implemented, this paper fails to show its effectiveness. PLOS One audience would like to read about how the new features would help them, both qualitatively and quantitatively compared with PubMed. Some features are truly unavailable on PubMed or other literature search engine, the authors did not quantify how the features can create results which are not available elsewhere.
This paper could have been more persuasive with more evidences and statistics.
Specifically I'd like to see the following additional information:
1. Experiments on important words/phrases: from Anne O'tate web log, find some examples and show how Important scores make sense in determining importance of words/phrases.

We have previously described Important Words in two papers and it is not one of the new functionalities emphasized in the current paper (although we have updated its display to a minor extent). Important words and phrases is shown in figures 4 and 5 for a specific search (Alzheimer and treatment) where the sense of the displayed words and phrases is hopefully self-evident.

2. Due to no disambiguation on author names, how much of Author count is accurate.

We have now clarified that the Author Count simply counts the number of authors on each single paper, NOT the author names. If a paper is written by “John Smith and John Smith”, the author count on that paper is 2.

3. How effective 'Mine the Gap!' can find gaps and predict future research lines.

This is cited in our previous publication and discussed there.

4. In Citation Cloud, bibliographically coupled articles may include some cases which are not based on similarity, how often such cases happened and how to minimize their damage to the results.

We have now clarified that bibliographically coupled (BC) articles are related in respect to partially shared reference lists , and very well may not be textually similar. This is actually a good thing, since BC and text similarity are two complementary types of similarity. We have now added citations to people who have discussed and studied this before.
