# Supplementary material for: Anne O’Tate: Value-added PubMed search engine for analysis and text mining
Source: PLoS One. 2021 Mar 8;16(3):e0248335. doi: 10.1371/journal.pone.0248335 (PMC7939269; doi:10.1371/journal.pone.0248335)
Supplement: Attachment — Submitted filename: Response to reviewers after second revision 1120.docx [file pone.0248335.s002.docx]

**Response to reviewers after second revision 1120**

Reviewer #2: One of the items in the major review was not addressed properly. The authors did not provide a proper use case in the discussion, but just hypotetical examples of how the tool could potentially be used in this or that situation. I meant a proper biomedical use case, e.g., looking for a treatment for disease X, and the various steps of how the searching was done in the tool and new knowledge has been found, etc. And that when doing such search in a similar way in PubMed, proving that no similar results could be obtained.

Thank you for this comment. **In the revised version, we now added text to the Discussion** **to indicate more clearly three practical use cases that would be easy in Anne but impossible in PubMed.** Anne O’Tate is designed to be extremely broad in adding value for a wide range of use cases; many of our features do not exist in PubMed and could not be conducted via PubMed searches. For example, our progressive drill-down architecture, our expand button, Important Words, Important Phrases, author count, predicted publication types, clustered by topic, important MeSH pairs, Mine the Gap! and Citation Cloud are all unique to Anne. We already indicated how these would be used and why, in the Results section.

Reviewer #4: This version provides numerous additional information. A user case is added to help understand how to use this web site and interpret the results. An impressive addition to the publication types certainly increases the value of the service.
The following are some issues with the draft:
1. The draft mentioned machine-learning based model to predict publication type. Please provide some details about the model.

**In the revised version, we have now added the following description of the model:** Briefly, positive training sets were collected for each of 50 publication types or study designs by taking a random sample of PubMed articles that were indexed with that category (publication type [Publication Type] or study design [MeSH term: no expansion]). We represented each article as a multi-dimensional vector of features taken from title, abstract, and metadata; among the features were unigrams and bigrams and several text similarity representations (paragraph2vec and our own implicit text similarity representation). Then, each publication type or study design was summarized as a single vector by averaging the vectors of the individual articles in that category. The vector distances of a given PubMed article to each of the category vectors was used to predict the probability p that it belongs to one or more of the categories. To implement the model in the Publication Types button in Anne O’Tate, we set a different threshold value of p for each category (having the highest F1 performance on a test set) and display articles under “Additional Predicted” if their predictive score p is over threshold but they lack NLM indexing for that category.

2. As pointed by other reviewers, when affiliation button was tested, most results showed similar mixture of country, city, institution, department names. A user of this button expects to see a list of entities at the same level, for example, a list of countries or a list of universities or institutions. For the query ‘Alzheimer AND treatment’, affiliation button retrieves more than 1, 000 pages of results. Without a search function, it is hard for the users to find the one they are interested in. Current design confused users and it needs more work to be useful.

We understand that the reviewer would like to see an entirely different architecture for the Affiliations button. **However, this button is not newly described in the present paper. Rather, it has been implemented for 12 or so years and was described in our previous paper.** We have neither had any user complaints nor have encountered use cases where we needed to re-engineer this button. We will take the reviewer’s advice to heart for future evolution of the system, but we do not feel that changing the Affiliations button should be a precondition for publishing the present paper.

3. Topic button simply uses MeSH terms to represent topic classification. Although a unified topic system is convenient and consistent, it is very small granularity. As a result, the same Alzheimer query returns more than 490 pages of results. Again, without a search function, the results are simply overwhelming for users.

**In the revised version, we have now clarified that because the MeSH terms are listed in order of document frequency, and because the top twenty terms are found on the first page, the most prevalent terms are immediately visible to the user,** though one can scroll to later pages to view the less prevalent terms if desired. Most of the 490 pages represents a long tail of MeSH terms found in only one article, hence are not very important at all.

4. Figure 2 displays some unicode characters incorrectly, although the website shows them correctly. Please update this figure.

The reviewer has sharp eyes! **We now made a new figure 2 that does not show incorrect Unicode characters.** (By the way, the Unicode mapping is a rather subtle issue. The retrieval of articles from PubMed does handle Unicode characters, but a few of the downstream functions involve mapping author names to normalized ascii formats (i.e., in the Authors button and in links out to the Author-ity tool) and so ascii is still displayed in a few places deep within Anne O’Tate. Users can manually replace the ascii characters with Unicode but that is not necessary for proper functioning.)
