# Supplementary material for: Anne O’Tate: Value-added PubMed search engine for analysis and text mining
Source: PLoS One. 2021 Mar 8;16(3):e0248335. doi: 10.1371/journal.pone.0248335 (PMC7939269; doi:10.1371/journal.pone.0248335)
Supplement: Attachment — Submitted filename: Response to Reviewers 0121.docx [file pone.0248335.s003.docx]

Dear Dr. Islamaj,

Thank you for allowing me to revise the paper a third time. I hope that you will be able to make a decision without returning it for further reviews. I have made a good faith effort to satisfy the concerns of both reviewers.

**Reviewer #4: Most issues have been addressed or explained.
I will make a small recommendation to the topic classification functionality. Currently the articles from different topics are displayed without the number of articles, while other buttons (Journals, pub types) display number of articles. It's good information for the user to know when they search.
I understand it may take time to implement but I really like to have this feature as a user.**

Thank you for your suggestion. We have now implemented this functionality as requested by the reviewer.

**Reviewer #2: Regarding my request for a proper use case, it hasn't been addressed by the authors. They simply listed three hypothetical, high-level situations, but they did not design a proper use case. The tool has been available since more than 10 years, but they could not prove, by citing a reference, that it has been used by other research groups to support solving any particular task. This was a request from me that the authors did not address. Therefore, I think that properly describing a use case which (somehow) prove the utility of the tool is crucial to be sure that this new version is really necessary and that the task could not be solved using other existing tools.**

As much as we would like to satisfy the reviewer, I think there is some misunderstandings that need to be clarified.

The second reviewer feels, in this and the previous review, that we should prove that Anne O’Tate can find biomedical information which cannot be found directly through PubMed searches alone (e.g. new treatments for a given disease) -- and that other biomedical researchers have used and cited Anne as having found such unique information. However, the original Anne O’Tate paper never made such claims, nor is that the intent of the original tool. The original tool was aimed at summarization, drill-down and mining of sets of articles retrieved from PubMed. It is NOT a literature-based discovery tool like Arrowsmith. Therefore, the type of documentation that the reviewer is asking for is not appropriate.

In the new version of Anne O’Tate that we present in the submitted manuscript, we do indeed introduce several NEW kinds of functionalities, that do find information that cannot be retrieved directly through PubMed searches (for example, author count, predicted publication types, and co-citations and bibliographically coupled articles in the citation cloud). Each of these is described and documented with specific examples. However, since these are brand-new and just now being reported, one cannot expect that these new functions have yet been used or cited by other research groups. Also, since these new functions are retrieving bibliographic rather than biomedical information, they are not likely to produce new knowledge such as new suggested treatments. (Though millions of people use search engines such as PubMed or Google Scholar to assist them, I do not believe that anyone has cited the use of these search engines in their research papers.)

In the final version of this ms., we DID cite references to show that others have reviewed and inspected Anne O’Tate, both from the technical as well as user points of view, and found it to have value for people carrying out literature searches:

“However, Anne O’Tate remains unique both in supporting progressive drill-down, and in its range of advanced mining options. To date, the original Anne O’Tate paper [1] has been cited 57 times according to Google Scholar (excluding self-citations) and 16 times in PubMed Central. In 2017, Engwall reviewed Anne O’Tate from the standpoint of users, especially librarians [8]. He critiqued its slow performance for large queries (which we have since fixed by moving to a different server and adding memory) and its bare-bones interface (which we deliberately prefer as a design choice), but concluded that “Anne O’Tate provides an excellent tool set for searching PubMed. Its data mining tools provide a variety of dynamic content analysis that can be of great use in identifying relevant search terms and bibliometrics.” [8].

I hope that this good-faith effort will suffice for publishing this manuscript.
